# Protein Amount, Quality, and Physical Activity

**DOI:** 10.3390/nu13113720

**Published:** 2021-10-22

**Authors:** Katsumi Iizuka

**Affiliations:** 1 The Department of Clinical Nutrition, Faculty of Medicine, Fujita Health University, Toyoake 470-1192, Japan; katsumi.iizuka@fujita-hu.ac.jp; 2 Yutaka Seino Distinguished Center for Diabetes Research, Kansai Electric Power Medical Research Institution, Kobe 650-0047, Japan

Diet composition determines the risk of obesity, cardiovascular disease, malignant tumors, and type 2 diabetes mellitus. To suggest an appropriate diet for patients, it is essential to consider both the amount and quality of macronutrients. In animal studies, a high sucrose, high fructose, or fat-rich diet causes obesity, fatty liver, and insulin resistance [1,2,3]. These effects are consistent with the results of a knockout mouse study using nutrient-sensing transcription factors [4]. A high-protein diet induces body weight and fat mass loss by reducing energy intake and expenditure. In a human study, the amounts of carbohydrates consumed were nonlinearly associated with mortality, including cardiovascular diseases. Concerning dietary fibers, the intake of vegetables and fruits is negatively associated with the risk of type 2 diabetes mellitus. Some studies have reported that the total level of protein intake is positively associated with all-cause mortality, indicating a harmful association between animal protein and CVD mortality [5]. Moreover, the nutritional intervention for protein intake is needed for the management and prevention of sarcopenia. Thus, diet composition is an important determinant affecting mortality and lifetime activity.

Recently, Fanelli reported a relationship between nutrient intake, diet quality, mortality, and functional limitations for 23,487 patients (>31 years old) with diabetes mellitus (a 2005–2016 National Health and Nutrition Examination Survey (NHANES) analysis) [6]. The patients with a lower protein intake (0.8 mg/kg of total bodyweight) consumed more carbohydrates and added sugars. In addition, the patients with a lower protein intake consumed less fat, including saturated and monounsaturated fats; vitamins (choline, vitamin B12, vitamin C, vitamin D, and vitamin K); phosphorus; and some minerals (zinc, magnesium, selenium, and sodium). Using the Healthy Eating Index 2015 (HEI-2015), a marker for total diet quality, the authors also estimated the total diet quality. In patients with a low protein intake, the HEI-2015 scores for the total number of consumed vegetables; wholegrains; dairy; and added sugars were much lower than those of the patients who met their protein recommendations. Moreover, patients with a lower protein intake showed a higher mean number of functional limitations. These results suggest that a higher level of protein intake is positively associated with the quality of diet and physical activity [6]. This study reveals that lower protein intake promotes a worse quality of physical activity in diabetic patients [6].

Protein consumption is negatively associated with the incidence of sarcopenia in diabetes mellitus [7]. This can be due to insulin resistance in diabetic patients. Insulin resistance may cause a decrease in amino acid utilization and protein synthesis in the muscles and thereby reduce muscle atrophy and physical activity. Adequate protein consumption is necessary to prevent the incidence of sarcopenia [6,7]. In contrast, many studies have reported that a low-protein, high-carbohydrate diet is beneficial for reducing mortality [8]. A high-protein, low-carbohydrate diet is also harmful to longevity and metabolic health [8]. In flies and mice, diets with a higher carbohydrate to protein ratio promoted insulin sensitivity and longevity [8]. In humans, a high-protein, low-carbohydrate diet was also associated with increased mortality rates [8]. In contrast, a diet with high protein and fat amounts benefitted the reproductive capacity in flies and mice [8]. Moreover, a high protein intake was associated with decreased levels of overall mortality and cancer mortality in the population over 65 years, but a 5-fold increase in diabetes mortality rates across all ages [9]. These results suggest that the amount of protein consumption should be changed in response to the aim (longevity, cardiovascular disease, diabetes, and sarcopenia) and age of the subject (<65 years old vs. ≥65 years old) [9].

The quality of protein is also an essential factor affecting the mortality rate associated with a diet. A high-animal-protein, low-carbohydrate diet was associated with higher all-cause mortality rates and cardiovascular and cancer mortality [10]. In contrast, a high-vegetable-protein, low-carbohydrate diet was associated with lower all-cause mortality rates and cardiovascular mortality [10]. In contrast, plant-based protein sources tended to have lower levels of essential AA (EAA) ratios than nonessential AAs (NEAAs) [11]. Therefore, the muscle protein synthetic responses to the ingestion of plant-based proteins were weaker than those of animal-based proteins [11]. In this study, patients with a lower intake of protein favored fatty and processed meats rather than plant or seafood-based proteins [6]. There are many differences in the content and composition of essential amino acids between various plant- and animal-based proteins [12]. Increasing the ratio of plant-based protein to animal-based protein could prevent cancer, cardiovascular disease, diabetes, and sarcopenia. Therefore, referring to the amino acid score of food and combining various foods is essential.

In conclusion, the education for patients with diabetes has been focused on the importance of carbohydrates and added sugars. This study provides evidence that indicates that a lower protein intake is associated with poor diet quality and physical limitations. A poor diet quality may be dependent on the lack of opportunity for education and socioeconomic circumstances. When selecting a protein source, it is essential to consider not only the amount of protein that should be consumed but also the quality of protein concerning the plant-based to animal-based ratio and amino acid score. Further clarification of the molecular mechanisms affecting the amount and quality of protein are also necessary to decide the protein intake recommendation for a healthy lifestyle.
